# Sex-specific hippocampal connectivity markers in mild cognitive impairment

**DOI:** 10.18632/aging.204660

**Published:** 2023-04-10

**Authors:** Yuan Yang, Andriy Yabluchanskiy

**Affiliations:** 1 Neural Control and Rehabilitation Laboratory, Stephenson School of Biomedical Engineering, University of Oklahoma, Tulsa, OK 74104,USA; 2 Department of Rehabilitation Sciences, The University of Oklahoma Health Sciences Center, USA; 3 Department of Physical Therapy and Human Movement Sciences, Northwestern University, Chicago, IL 60611, USA; 4 Vascular Cognitive Impairment and Neurodegeneration Program, Oklahoma Center for Geroscience and Healthy Brain Aging, Department of Neurosurgery, The University of Oklahoma Health Sciences Center, USA

**Keywords:** sex difference, mild cognitive impairment, hippocampus, fMRI, functional connectivity

**Commentary on:** Williamson J, et al. Sex differences in brain functional connectivity of hippocampus in mild cognitive impairment. Front Aging Neurosci. 2022; 14:959394. https://doi.org/10.3389/fnagi.2022.959394. PMID:36034134

Alzheimer’s disease (AD) is associated with a number of significant sex differences. It is well documented that about two-thirds of those with AD are women; men and women with AD exhibit different cognitive and psychiatric symptoms with more severe manifestation observed in women; and women show a faster cognitive decline after the diagnosis of mild cognitive impairment (MCI), the prodromal stage to dementia, toward the AD [1, 2].

**Figure 1:**
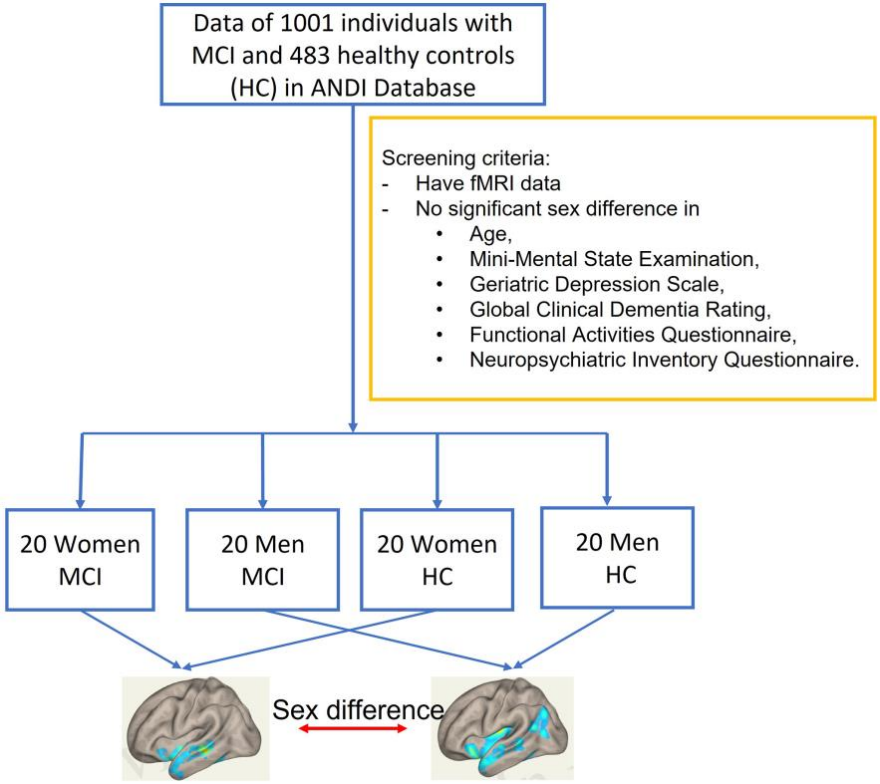
The general scheme of data analysis in Williamson, et al. (2022) [3].

Recently, the collaborative team of Dr. Yang reported that functional connectivity of the hippocampus to the precuneus cortex and brain stem was significantly stronger in men than in women in MCI [3]. This study was conducted on the functional Magnetic resonance imaging (fMRI) data collected through the Alzheimer’s Disease Neuroimaging Initiative (ADNI, adni.loni.usc.edu). ADNI is a publicly accessible database that measures the progression of MCI toward early AD. The analysis team conducted a screening on 1001 individuals with MCI and 483 healthy controls to select a total of 80 subjects’ data including 40 women and men with MCI (*n* = 20 per group), and cognitively normal women and men (*n* = 20 per group). The screening ensured there was not a statistically significant sex difference in age, Mini-Mental State Examination, Geriatric Depression Scale, Global Clinical Dementia Rating, Functional Activities Questionnaire, and Neuropsychiatric Inventory Questionnaire. A seed-to-voxel analysis was conducted to examine hippocampal connectivity to other regions in the brain. The general scheme of this research is demonstrated in Figure 1. Results from this study demonstrate that, in MCI, women showed significantly reduced connectivity of the right or left hippocampus to the left or right precuneus cortex, when compared to men. There was also a sex difference identified in the hippocampal connectivity to the brain stem.

Both hippocampus and precuneus cortex are highly associated with the process of memory, spatial function, and navigation [4]. The reduced functional connectivity between the hippocampus and precuneus cortex likely explains why females may have an accelerated progression of MCI to AD. In addition, previous studies have reported that the functional connectivity of midbrain structures of the brain stem differs in individuals with AD and MCI [5]. These structures were associated with the occurrence of neuropsychiatric symptoms of AD [6]. The sex difference in hippocampal connectivity to the brain stem, therefore, may also be associated with sex-specific progress of the cognitive impairment stage.

In summary, the findings in this work extend current understanding of the role of the hippocampus-precuneus cortex and hippocampus-brainstem connectivity in sex differences in MCI. Understanding these sex differences in neuro-pathophysiology may aid in the future development of sex-specific precision rehabilitation strategies to manipulate hippocampal-precuneus cortex and hippocampal-brainstem connectivity to stop the progression of MCI to AD. The findings provide the rationale for sex-specific interventions such as neuro-navigation guided, targeted non-invasive brain stimulation that is currently explored by the same team.
